# Mitochondrial Dysfunction in Degenerative Diseases

**DOI:** 10.3390/cells11091546

**Published:** 2022-05-05

**Authors:** Julio Cesar Batista Ferreira

**Affiliations:** Department of Anatomy, Institute of Biomedical Sciences, University of Sao Paulo, Sao Paulo 05508-070, Brazil; jcesarbf@usp.br

Mitochondria are major intracellular hubs distributed throughout the cell that play a key role in the spatiotemporal coordination and propagation of signalling events, ensuring that homeostasis is met at baseline or under environmental pressure. These organelles are a major source of biomolecules such as ATP, peptides, phospholipids, and reactive molecules, which can directly control a plethora of intracellular events including, but not limited to, nuclear gene expression, ion homeostasis, protein synthesis/trafficking/degradation, protein–protein interaction, autophagy, cell cycle, and cell death [1]. As expected, genetic disruption of mitochondrial function, termed mitochondriopathy or mitochondrial disease, compromises its ability to orchestrate an extensive response against metabolic or functional oscillations triggered by a wide range of acute and chronic stress conditions. Under this scenario, a lack of mitochondria response to environmental pressure has a detrimental effect on both healthspan and lifespan.

Mitochondrial dysfunction plays a main role in the establishment and progression of a wide spectrum of degenerative diseases including, but not limited to, heart failure, hypertension, obesity, diabetes, liver failure, kidney failure, Alzheimer’s disease, Parkinson’s disease, and Huntington disease [2]. Therefore, the activation of mechanisms of surveillance and quality control responsible for the maintenance of mitochondrial functionality and integrity is critical to tackling such diseases. Mitochondrial quality is fine-tuned by a myriad of interconnected systems, including (1) enzymatic and non-enzymatic elements capable of fighting oxygen-mediated mitochondrial toxicity; (2) mitochondrial proteases and chaperones responsible for the maintenance of mitochondrial proteostasis; and (3) a multilayer network of proteins involved in the control of mitochondrial morphology, location, and number.

As shown by the original research, brief communication, and review articles assembled for this Special Issue on “Mitochondrial Dysfunction in Degenerative Diseases”, a better understanding of regulatory processes involved in mitochondrial bioenergetics, surveillance, and quality control mechanisms in health and disease will guide the development of novel approaches for diagnostics and therapeutics against degenerative diseases. This Special Issue consists of four original articles, one brief communication, and two review articles that broaden our understanding of the regulatory processes associated with mitochondrial biology in health and disease. In turn, these manuscripts bring new insights into mitochondria as intracellular hubs as well as propose novel targets for diagnostics and treatment of degenerative diseases. They cover the topics of (1) redox signalling and oxidative stress, (2) mitochondrial proteostasis, (3) adrenergic signalling and mitochondria, (4) mitochondrial dynamics, and (5) mitochondrial bioenergetics.

One article in this Special Issue focused on the interplay between the beta adrenergic signalling pathway and mitochondria bioenergetics in skeletal muscle during exercise. Voltarelli et al. demonstrated that exercise induces β_2_-adrenergic receptor activation, which favours the accumulation of functional elongated mitochondria in skeletal muscle from mice [3]. As a proof of concept, either a pharmacological blockade of β_2_-adrenergic receptor or the inhibition of its downstream signalling pathway through protein kinase A (PKA) abolished the mitochondrial effects of exercise or the β-adrenergic receptor agonist (isoproterenol) both in vivo and in myocytes in culture. These findings uncover the β_2_-adrenergic receptor signalling pathway as a key contributor to the mitochondrial plasticity and bioenergetics in skeletal muscle during exercise. However, the mechanisms of action by which β_2_-adrenergic receptor stimulation improves mitochondrial function and morphology, including the identification of major mitochondrial effectors, still need to be determined. In another article, Portz et al. used a transgenic mouse model expressing a familial Parkinson disease-linked α-synuclein mutation (A53T) to study the effect of α-synucleinopathy on mitochondrial dynamics in neurons [4]. The authors reported that mitochondrial drp1 levels are reduced in neurons from transgenic Parkinson’s disease, which are paralleled by the accumulation of enlarged mitochondria compared with control mice. These findings strengthen the role of mitochondrial fission in neurodegenerative diseases.

Another article in this Special Issue discussed the role of voltage-dependent anion channel (VDAC) in mitochondrial dysfunction and diseases [5]. VDAC is located in the outer mitochondrial membrane and has two conductance states (open and close), which control the mitochondrial permeability of ADP/ATP, calcium homeostasis, and apoptosis. Three reported structures of VDAC in the open-state conformation provided novel insights about its role in regulating metabolite transport and the mitochondrial permeability transition pore, which contribute to different degenerative diseases. Further information on the closed state of VDAC is needed, as pointed out by the authors. Another review article highlighted the most recent advances in both biochemical and structural features of translocase of the outer membrane (TOM) complex and its mitochondrial protein import machinery [6]. Pitt et al. comprehensively described the role of the TOM complex as a gateway for proteins that control mitochondrial function under health and disease and discussed some strategies to develop structure-based therapeutics targeting TOM complex to treat degenerative diseases.

In another article, Parada et al. demonstrated that intracellular peptides generated during proteasome-mediated protein degradation play a key role in regulating protein–protein interaction [7]. A cell-permeable synthetic peptide was designed based on a fragment of the FKBP12 (FK506-binding protein) polypeptide sequence produced during proteasomal degradation. This short peptide, termed VFD, inhibited rapamycin-induced interaction between PKBP and FRB (FKBP12-rapamycin binding domain) in vitro. Considering that mitophagy and mitochondrial function are directly regulated by the PKBP-FRB complex, it is expected that such peptides might have a therapeutic value in treating degenerative diseases. In fact, a similar approach was recently used to fix mitochondrial dynamics using rationally designed synthetic short peptides targeting protein–protein interactions, which was sufficient to improve mitochondrial health and heart failure outcome in rats [8].

Mitochondrial dysfunction has been recently linked to the pathophysiology of pain in both rodents and humans [9,10]. Indeed, two out of three people with mitochondrial disease have chronic pain. Unfortunately, pre-clinical models mimicking pain in a dish are not robust enough to recapitulate the intracellular signature of pain, including mitochondria dysfunction, therefore limiting the discovery/validation of novel targets and screening of new molecular entities to treat pain. Bufalo et al. established a preclinical model using collagen-derived advanced glycation end-products to activate human sensory-like neurons in culture, which was reduced by morphine [11]. This model can be used to explore the mitochondrial contribution to neuronal activation under pain-related conditions in a dish as well as become a platform for high content analysis to identify hits with analgesic properties. Finally, McCarty et al. speculated about the protective role of magnesium supplementation in counteracting some of the detrimental effects of elevated intracellular phosphate levels, which might have a potential to tackle oxidative stress in degenerative cardiovascular diseases [12]. I hope that this Special Issue provides novel insights and nurtures new ideas into mitochondrial function and how it drives biology under health and degenerative disease conditions.
